# Effects of dobutamine on intestinal microvascular blood flow heterogeneity and O_2_ extraction during septic shock

**DOI:** 10.1152/japplphysiol.00886.2016

**Published:** 2017-03-23

**Authors:** Gustavo A. Ospina-Tascón, Alberto F. García Marin, Gabriel J. Echeverri, William F. Bermudez, Humberto Madriñán-Navia, Juan David Valencia, Edgardo Quiñones, Fernando Rodríguez, Angela Marulanda, César A. Arango-Dávila, Alejandro Bruhn, Glenn Hernández, Daniel De Backer

**Affiliations:** ^1^Department of Intensive Care Medicine, Fundación Valle del Lili, Universidad ICESI, Cali, Colombia;; ^2^Universidad del Valle, Escuela de Ciencias Básicas, Cali, Colombia;; ^3^Departamento de Medicina Intensiva, Pontificia Universidad Católica de Chile, Santiago, Chile; and; ^4^Intensive Care Department, CHIREC Hospitals, Université Libre de Bruxelles, Brussels, Belgium

**Keywords:** microcirculation, microcirculatory blood flow, villi perfusion, oxygen extraction ratio, oxygen consumption, gut mucosal perfusion

## Abstract

Derangements of microvascular blood flow distribution might contribute to disturbing O_2_ extraction by peripheral tissues. We evaluated the dynamic relationships between the mesenteric O_2_ extraction ratio ($\text{mes-E}{\text{R}}_{{\text{O}}_{2}}$) and the heterogeneity of microvascular blood flow at the gut and sublingual mucosa during the development and resuscitation of septic shock in a swine model of fecal peritonitis. Jejunal-villi and sublingual microcirculation were evaluated using a portable intravital-microscopy technique. Simultaneously, we obtained arterial, mixed-venous, and mesenteric blood gases, and jejunal-tonometric measurements. During resuscitation, pigs were randomly allocated to a fixed dose of dobutamine (5 µg·kg^−1^·min^−1^) or placebo while three sham models with identical monitoring served as controls. At the time of shock, we observed a significant decreased proportion of perfused intestinal-villi (villi-PPV) and sublingual percentage of perfused small vessels (SL-PPV), paralleling an increase in $\text{mes-E}{\text{R}}_{{\text{O}}_{2}}$ in both dobutamine and placebo groups. After starting resuscitation, villi-PPV and SL-PPV significantly increased in the dobutamine group with subsequent improvement of functional capillary density, whereas $\text{mes-E}{\text{R}}_{{\text{O}}_{2}}$ exhibited a corresponding significant decrease (repeated-measures ANOVA, *P* = 0.02 and *P* = 0.04 for time × group interactions and intergroup differences for villi-PPV and $\text{mes-E}{\text{R}}_{{\text{O}}_{2}}$, respectively). Variations in villi-PPV were paralleled by variations in $\text{mes-E}{\text{R}}_{{\text{O}}_{2}}$ (*R*^2^ = 0.88, *P* < 0.001) and these, in turn, by mesenteric lactate changes (*R*^2^ = 0.86, *P* < 0.001). There were no significant differences in cardiac output and systemic O_2_ delivery throughout the experiment. In conclusion, dynamic changes in microvascular blood flow heterogeneity at jejunal mucosa are closely related to the mesenteric O_2_ extraction ratio, suggesting a crucial role for microvascular blood flow distribution on O_2_ uptake during development and resuscitation from septic shock.

**NEW & NOTEWORTHY** Our observations suggest that dynamic changes in the heterogeneity of microvascular blood flow at the gut mucosa are closely related to mesenteric O_2_ extraction, thus supporting the role of decreasing functional capillary density and increased intercapillary distances on alterations of O_2_ uptake during development and resuscitation from septic shock. Addition of a low-fixed dose of dobutamine might reverse such flow heterogeneity, improving microcirculatory flow distribution and tissue O_2_ consumption.

## Abstract (precis)

Our observations suggest that dynamic changes in the heterogeneity of microvascular blood flow at the gut mucosa are closely related to mesenteric O_2_ extraction, thus supporting the role of decreasing functional capillary density and increased intercapillary distances on alterations of O_2_ uptake during development and resuscitation from septic shock. Addition of a low-fixed dose of dobutamine might reverse such flow heterogeneity, improving microcirculatory flow distribution and tissue O_2_ consumption.

one of the most notable characteristics of the inflammatory response during sepsis in both humans and animal experimental models is its deleterious effect on microcirculation. These microcirculatory blood flow alterations are mainly characterized by decreased functional capillary density and increased heterogeneity leading to blood flow misdistribution (1, 10, 18, 23, 34), tissue hypoperfusion, and the subsequent development of multiple organ dysfunction (48). It was initially proposed that a mismatch of O_2_ demand to supply could impair O_2_ utilization by tissues (26, 40, 41). This notion was reinforced by mathematical models suggesting that heterogeneity of O_2_ delivery could decrease tissue O_2_ extraction (49). In fact, evidence coming from ex vivo intestinal tissue in endotoxemic pigs revealed the importance of increased heterogeneity in microcirculatory transit times on the impairment of O_2_ extraction (21). The nature of such experiments, however, hindered the temporal variations of heterogeneity of flow during the development of, and resuscitation from, septic shock. Other experimental data suggested that the misdistribution of microvascular blood flow leads to O_2_ capillary extraction derangements during sepsis (11) such that heterogeneous flow cessation of individual capillaries could determine O_2_ supply dependence during the most severe cases of septic shock (16).

The gastrointestinal tract is particularly prone to being affected during low flow states (9, 44), and it has been hypothesized that improvement in splanchnic perfusion might prevent the progression of shock. Thus, some synthetic catecholamines capable of increasing cardiac output and O_2_ delivery have been used aiming at reverting tissue hypoperfusion. Its impact on splanchnic circulation remains controversial, however, with apparent favorable (3, 33, 37, 39, 47), unchanged (35), or even negative effects (2, 13) on both splanchnic arterial flow and total microcirculatory blood flow at the intestinal mucosa. Despite these different effects on macro and total microvascular blood flow, some vasoactive amines and inodilators have been shown to promote favorable effects on intestinal oxygenation (14), although mucosal oxygenation might, in turn, be dissociated from splanchnic O_2_ delivery when some of them are infused (33, 43), which suggests the predominance of distributive alterations over total reductions in microcirculatory blood flow. Notably, microvascular blood flow distribution could potentially be modified by inodilators during septic shock (7, 42), although its effect during human septic shock remains controversial (12, 18).

We therefore proposed to evaluate the dynamic variations of the heterogeneity of blood flow distribution at the intestinal mucosa and its relation with the regional O_2_ extraction ratio during the development and resuscitation of septic shock in a model of fecal peritonitis subjected to hemodynamic goal-directed fluid resuscitation and randomly assigned to receive, or not, a low-fixed dose of dobutamine, hypothesizing that variations in microcirculatory heterogeneity are closely related to changes in regional O_2_ extraction independent of macrohemodynamic changes.

## METHODS

### 

#### Animal preparation.

Our institutional Animal Research Committee approved the present study (Res. 001–12). Fifteen female Landrace pigs (35–42 kg) were kept fasting for a 12-h period, with free access to water. After this preconditioning period, they were initially sedated with intramuscular injections of ketamine (5–10 mg/kg) and xylazine (0.1 mg/kg). Next, a venous access (Insyte Autoguard infusion therapy system) was inserted in the ear to facilitate the administration of medication and fluids. After an intravenous dose of propofol (2–4 mg/kg) and fentanyl (5 µg/kg), an endotracheal tube and mainstream volumetric capnography were placed (Infinity EtCO_2_ + respiratory mechanics module; Dräger Medical Systems). The animals were connected to mechanical ventilation (Servo 900C; Siemens, Solna, Sweden) in assist control mode, with a tidal volume of 12 ml/kg, and the volume per minute was adjusted to maintain arterial Pco_2_ at 38 ± 5 mmHg. Anesthesia was maintained throughout the experiment with midazolam (3–5 µg·kg^−1^·min^−1^), fentanyl (0.03–0.05 µg·kg^−1^·min^−1^), and propofol (50 µg·kg^−1^·min^−1^). Muscular paralysis was provided with pancuronium bromide (5 µg·kg^−1^·min^−1^) during the entire experiment. Neck vessels were accessed by direct dissection, and catheters were inserted in the carotid artery (Single lumen central venous 7-Fr catheter; Arrow International) to monitor arterial pressure and to enable blood sampling while the left internal jugular vein was used for resuscitation fluid infusion. We also placed a continuous-cardiac-output (CCO) pulmonary artery catheter (7.5-Fr, Edwards Swan-Ganz CCO; Baxter Edwards Critical Care, Irvine, CA) through the right internal jugular vein to measure pulmonary arterial pressures and continuous cardiac output and to enable the withdrawal of mixed-venous blood samples. Core temperature was continuously monitored using a thermistor in the pulmonary artery catheter, and external heating was used to maintain a central temperature of 36.5 ± 1°C. Continuous electrocardiographic, pulsioximetry, and invasive pressures were also monitored throughout the experiment (Drägger Infinity Vista XL; Drägger Medical System, Lübeck, Germany).

A midline laparotomy was performed, and a gas-tonometer catheter (TRIP Tonometry Catheter, 8-Fr; Tonometrics Division, Instrumentarium, Helsinki, Finland) was placed in the jejunal lumen at 50 cm beyond the Treitz angle for local tissue CO_2_ and pH_i_ measurements. A double-lumen catheter (2-lumen central venous 7-Fr gauge catheter; Arrow International) was inserted in the superior mesenteric vein through the splenic vein after splenectomy and local constriction with epinephrine, ensuring its position under echographic guide. An infusion with 0.9% saline solution at 5 ml/h through this catheter was provided during the experiment to ensure its permeability. Surgical cystostomy was also undertaken to monitor urinary output. A jejunum loop was exteriorized through the midline incision, and a small segment was opened along its antimesenteric border using an electrocautery. After baseline measurements, cecal ligation and puncture with 16-gauge needle followed by peritoneal contamination (1.5 g/kg of feces) were performed in experimental models (not for sham animals). After careful hemostasis, the abdominal contents were returned to the cavity, and the abdomen was closed, leaving out the jejunostomy loop, which was then covered with moistened compresses and an anti-adherent bag to avoid heat and fluid loss.

#### General monitoring.

Mean arterial pressure was monitored throughout the experiment, and pulse pressure variations (dPP) were calculated during the respiratory cycle as: PPinsp – PPexp/(PPinsp + PPexp/2) (Drägger Infinity Vista XL; Drägger Medical System). Mean pulmonary artery, central venous, and pulmonary arterial occlusion pressures were measured at the end of expiration and referenced to the midchest level. Cardiac output was continuously measured using the thermodilution principle with a thermal filament on the pulmonary artery catheter (Vigilance; Baxter Edwards Critical Care). A mainstream capnography and respiratory module (Dräger Medical Systems) was used to measure airway and alveolar dead-space fraction, end-tidal CO_2_, and complete respiratory mechanics.

#### Calculation of CO_2_ and O_2_ variables.

Simultaneous arterial (a), mixed-venous ($\bar{\text{v}}$), and mesenteric-venous (mes) blood samples were withdrawn at each measurement time point to determine blood gases, hemoglobin, and lactate concentrations (The Alere Epoc blood analysis system; Alere, Waltham, MA). Concurrent mucosal jejunal CO_2_ was measured by gas tonometry (Tonocap, Datex-Ohmeda; Tonometrics Division, Instrumentarium) as described elsewhere (4). O_2_ and CO_2_ parameters were calculated according to the following formulas:

$$\text{C}{\text{a}}_{{\text{O}}_{2}}=(\text{Hg}\times \text{S}{\text{a}}_{{\text{O}}_{2}}\times 1.34)+(\text{P}{\text{a}}_{{\text{O}}_{2}}\times 0.003)$$

$$\text{C}{\text{v}}_{{\text{O}}_{2}}=(\text{Hg}\times \text{S}{\text{v}}_{{\text{O}}_{2}}\times 1.34)+(\text{P}{\text{v}}_{{\text{O}}_{2}}\times 0.003)$$

$$\text{Da-}{\text{v}}_{{\text{O}}_{2}}=\text{C}{\text{a}}_{{\text{O}}_{2}}-\text{C}{\text{v}}_{{\text{O}}_{2}}$$

$$\text{D}{\text{o}}_{2}=\text{C}{\text{a}}_{{\text{O}}_{2}}\times \text{CO}$$

$$\overset{․}{V}{\text{o}}_{2}=\text{C}{\text{a}}_{{\text{O}}_{2}}-\text{C}{\text{v}}_{{\text{O}}_{2}})\times \text{CO}$$

$$\text{E}{\text{R}}_{{\text{O}}_{2}}=(\text{C}{\text{a}}_{{\text{O}}_{2}}-\text{C}{\text{v}}_{{\text{O}}_{2}})/\text{C}{\text{a}}_{{\text{O}}_{2}}$$

$$\text{mes-E}{\text{R}}_{{\text{O}}_{2}}=(\text{C}{\text{a}}_{{\text{O}}_{2}}-\text{Cvme}{\text{s}}_{{\text{O}}_{2}})/\text{C}{\text{a}}_{{\text{O}}_{2}}$$

$$\text{Mixed venous-to-arterial}{\;CO}_{2}\text{ difference }(\text{P}\overset{¯}{\text{v}}\text{-}{\text{a}}_{\text{C}{\text{O}}_{2}})=\text{P}{\overset{¯}{\text{v}}}_{\text{C}{\text{O}}_{2}}-\text{P}{\text{a}}_{\text{C}{\text{O}}_{2}}$$

$$\text{Mesenteric venous-to-arterial }{CO}_{2}\text{ difference }(\text{Pmes-}{\text{a}}_{\text{C}{\text{O}}_{2}})=\text{Cvme}{\text{s}}_{\text{C}{\text{O}}_{2}}-\text{P}{\text{a}}_{\text{C}{\text{O}}_{2}}$$

$$\text{Jejunum mucosal-to-arterial }{CO}_{2}\text{ difference }(\text{Ptis-}{\text{a}}_{\text{C}{\text{O}}_{2}})=\text{Pti}{\text{s}}_{\text{C}{\text{O}}_{2}}-\text{P}{\text{a}}_{\text{C}{\text{O}}_{2}}$$

where $\text{C}{\text{a}}_{{\text{O}}_{2}}$ and $\text{C}{\text{v}}_{{\text{O}}_{2}}$ are the arterial and venous O_2_ content, respectively; $\text{P}{\text{a}}_{\text{O}}{}_{{}_{2}}$ and ${\text{Pv}}_{{\text{O}}_{2}}$ represent the arterial and venous partial pressures, respectively, and CO represents cardiac output. $\text{E}{\text{R}}_{{\text{O}}_{2}}$ and $\text{mes-E}{\text{R}}_{{\text{O}}_{2}}$ represent the total and mesenteric O_2_ extraction ratio, respectively.

#### Microcirculatory measurements.

**Fig. 1.**
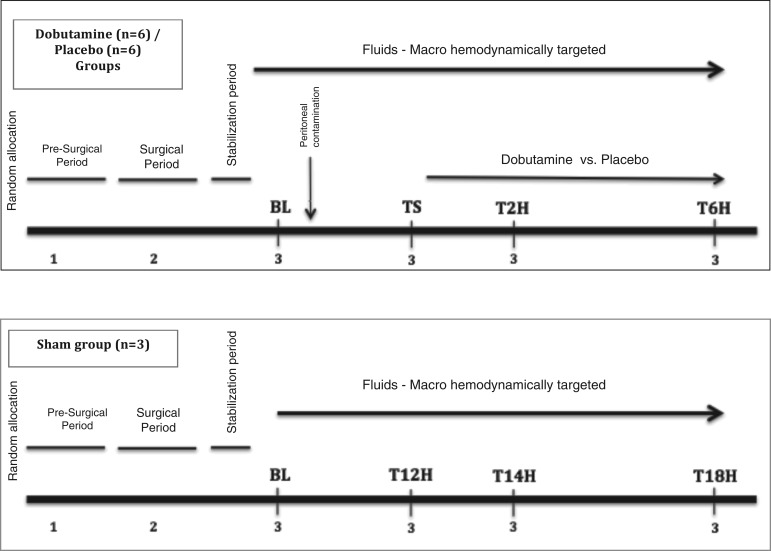
Experimental protocol. 1, Presurgical period (acclimatization-adaptation period, initial sedation, muscular paralysis, endotracheal intubation); 2, surgical period (catheters insertion: carotid and pulmonary artery, internal jugular vein, mesenteric vein; laparotomy: jejunal tonometer insertion; cystostomy; jejunal ostomy preparation); 3, serial measurements [arterial, mixed-venous, and mesenteric-venous blood sampling; jejunal tonometric measurements; acquisition of microcirculation images (jejunal mucosa, jejunal serosa, sublingual mucosa)]. BL, baseline; TS, time of shock; T2H, 2 h after starting resuscitation; T6H, 6 h after starting resuscitation.

We used the Sidestream dark-field (SDF) technique (Micro Scan; MicroVision Medical, Amsterdam, The Netherlands) to explore microcirculation at each measurement time point (Fig. 1). In all cases, images were acquired by the same operator (Ospina-Tascón) who remained blinded as to dobutamine or placebo use both during acquisition and the semiquantitative analysis of the video sequences. After careful removal of intestinal secretions by warm water and gentle aspiration, the SDF device was directly applied through the surgically prepared jejunostomy and on the antimesenteric opposite serosa surface to evaluate jejunal villi and serosa microcirculation, respectively, covering an intestinal segment of at least 15 cm. Meanwhile, sublingual microcirculation was assessed by SDF soft application to the lateral side of the tongue covering an area approximately of 4–6 cm from the tip of tongue after gentle removal of secretions with gauze. At each measurement time point (Fig. 1), five video sequences of 20 s each were acquired at five different points from the respective mucosa or adjacent serosa areas using a videocard (MicroVideo; Pinnacle System, Mountain Views, CA). These sequences of video were stored under a random number and later evaluated by two investigators blind to the origin of such sequences (Quiñones and Ospina-Tascón). For this semiquantitative analysis, we counted the number of villi in each image, and individual villi microcirculation was classified according to its predominant blood flow as either normal perfused (continuous blood flow), hypoperfused (intermittent or sluggish blood flow), or nonperfused (stopped blood flow). Thus, we calculated the percentage of villi with normal-perfused capillaries in each video sequence to finally report the mean of the five video sequences acquired at each time point. Meanwhile, for serosa and sublingual microcirculation evaluation, we used a cutoff value of 20 μm to classify the vessels as large or small. Microvessels with continuous flow were considered as normal, whereas sluggish, intermittent, and stopped flows were considered as abnormal. Serosa and sublingual microcirculatory blood flow were evaluated according to the consensus for the evaluation of microcirculation (8), and we also calculated the proportion of small-perfused vessels (<20 μm diameter), the heterogeneity index (HI), the total vascular density (all vessels), and the functional capillary density (i.e., the number of vessels <20 μm of diameter adequately perfused, per area unit). The intra- and interobserver variabilities were determined by using five sequences analyzed five times at 8-wk intervals by two observers (Quiñones and Ospina-Tascón). We calculated the intraobserver and interobserver coefficient of variability for both the total number of vessels and the proportion of perfused vessels.

#### Experimental protocol.

The experimental protocol is outlined in Fig. 1. After the initial preparation, a stabilization period of 30 min was ensured, and then baseline measurements (BL) were performed. Animals were subsequently randomly allocated to the dobutamine (*n* = 6), placebo (*n* = 6), or sham (*n* = 3) group. An independent laboratory staff member (not participating as an author in the present manuscript) was in charge of the randomization and preparation of these experimental infusions. Cecal ligation and puncture, followed by peritoneal contamination, was performed only in the dobutamine and placebo models. All animals received intravenous lactate Ringer fixed infusion at 3 ml/kg throughout the experiment. When hypotension was developed and it was not corrected by successive crystalloid boluses (at least 30 ml/kg), norepinephrine (NE) infusion was started and titrated to maintain mean arterial pressure (MAP) >65 mmHg. Once the NE dose remained stable for at least 30 min, time shock (TS) was declared, and a new set of measurements were obtained. After this point, resuscitation was conducted using successive fluid loads of crystalloid (at 10 ml/kg) guided by dynamic predictors of fluid responsiveness to optimize cardiac output. Concomitantly, fixed doses of dobutamine (5 µg·kg^−1^·min^−1^) or placebo (0.9% saline solution at isovolumetric dose) were infused throughout the experiment. A new set of measurements was performed at 2 (T2H) and 6 (T6H) h afterward. Sham animals were subjected to identical monitoring as the experimental groups. The timing used for measurements in the sham group was referenced to the median time required from peritonitis induction to fulfilling shock definition during the preexperimental standardization phase. Thus, T12H in the sham group was selected as the matched time for TS, so new measurements were performed 2 and 6 h later, thus constituting T14H and T18H, i.e., the equivalent to T2H and T6H in the experimental models (Fig. 1). Euthanasia was performed at the end of the experiment using Euthanex according to the local regulations for animal research.

#### Statistical analysis.

Data are reported as medians and interquartile ranges. After testing the sphericity assumptions, time, time-group interactions, and intergroup differences for the experimental groups were evaluated using the repeated-measures analysis of variance, with a subsequent Student-Newman-Keuls (SNK) test for multiple comparisons. The relationship between microcirculatory blood flow variables, lactate levels, and mesenteric O_2_ extraction ratio was tested using the Spearman Rho test, and the coefficient of determination (*R*^2^) was calculated to establish the strength of such associations. A *P* value ≤ 0.05 (2-tailed) was considered significant.

## RESULTS

### 

#### Systemic hemodynamics and O_2_ transport.

General hemodynamics, blood gases, and fluids/vasopressor use are presented in Table 1. Hypodynamic shock was developed 12 (10–14) h after the induction of peritonitis in the dobutamine (D group) and placebo (P group) models, with similar falls in cardiac output [91.2 (84.8–94.1) vs. 75.0 (68.2–95.1) ml·kg^−1^·min^−1^ for P and D group, respectively; *P* = 0.21] and systemic O_2_ delivery [13.4 (12.5–15.1) vs. 13.9 (10.9–15.0) ml·kg^−1^·min^−1^ for P and D groups, respectively; *P* = 0.88] (Table 1). Subsequently, cardiac output and systemic O_2_ delivery exhibited a similar improvement in the D and P groups at T2H and T6H (repeated-measurements analysis, *P* = 0.86 and 0.84 for time × group interactions, and *P* = 0.51 and 0.56 for intergroup differences for CO and Do_2_, respectively) (Table 1).

**Table 1.** Hemodynamics, blood gases, and O_2_/CO_2_ parameters

					Intragroup Difference		
	Baseline	TS	T2H	T6H	Time effect	Time × group effect	Intergroup Difference
*Hemodynamic variables*							
HR, beats/min							
Placebo	90 (81–130)	154 (131–180)	155 (153–168)	164 (132–174)	0.01	0.99	0.34
Dobutamine	97 (87–128)	187 (113–205)	156 (142–189)	172 (146–197)			
Sham	146 (140–151)	108 (96–120)	99 (98–100)	70 (54–85)			
CO, ml·kg^−1^·min^−1^							
Placebo	122.9 (111.8–138.2)	91.2 (84.8–94.1)	125.7 (120.6–138.2)	138.2 (128.6–145.5)	<0.001	0.86	0.51
Dobutamine	100.0 (90.9–120.0)	75.0 (68.2–95.1)	106.8 (90.9–144.1)	123.2 (104.5–152.7)			
Sham	104.3 (100.0–108.6)	108.7 (108.6–108.8)	108.7 (105.7–111.8)	113.0 (111.8–114.3)			
MAP, mmHg							
Placebo	102 (92–114)	70 (69–77)	71 (69–82)	65 (63–97)	0.001	0.18	0.54
Dobutamine	100 (88–105)	81 (77–87)	67 (65–71)	77 (60–82)			
Sham	97 (87–106)	100 (81–118)	102 (100–105)	129 (123–135)			
CVP, mmHg							
Placebo	12 (7–21)-	13 (10–15)	13 (11–18)	14 (12–22)	0.15	0.98	0.29
Dobutamine	10 (5–14)	9 (8–10)	11 (10–12)	12 (10–15)			
Sham	14 (13–15)	15 (13–16)	13 (12–15)	15 (11–18)			
Temperature, °C							
Placebo	35.8 (34.3–36.4)	38.1 (35.1–38.3)	37.8 (34.3–38.1)	36.8 (33.8–38.4)	0.02	0.76	0.38
Dobutamine	35.9 (34.9–37.0)	38.2 (37.1–39.8)	37.0 (36.4–37.6)	36.6 (36.3–37.3)			
Sham	34.7 (33.8–35.6)	34.6 (33.5–35.7)	35.1 (34.1–36)	34.5 (33.1–35.6)			
*Blood gases, lactate, and O_2_ variables*							
pH (arterial)							
Placebo	7.38 (7.37–7.40)	7.34 (7.28–7.36)	7.31 (7.24–7.41)	7.20 (7.10–7.42)	0.002	0.11	0.55
Dobutamine	7.38 (7.38–7.39)	7.27 (7.21–7.28)	7.20 (7.14–7.26)	7.29 (7.27–7.32)			
Sham	7.39 (7.38–7.40)	7.42 (7.40–7.43)	7.41 (7.39–7.42)	7.42 (7.40–7.45)			
$\text{P}{\text{a}}_{\text{CO}}{}_{{}_{2}}$, mmHg							
Placebo	34.6 (32.9–37.5)	40.1 (35.0–40.2)	37.6 (36.2–42.4)	38.6 (37.2–40.3)	0.01	0.15	0.83
Dobutamine	29.9 (29.5–33.2)	41.7 (36.3–50.5)	42.2 (41.6–46.3)	39.1 (36.0–39.8)			
Sham	34.1 (33.6–34.5)	34.7 (34.2–35.1)	34.7 (34.2–35.1)	35.0 (34.6–35.3)			
$\text{Pv-}{\text{a}}_{\text{C}{\text{O}}_{2}}$, mmHg							
Placebo	3.0 (2.7–4.2)	12.0 (11.0–15.5)	11.7 (11.6–16.0)	9.9 (9.3–14.1)*	<0.001	0.06	0.05
Dobutamine	4.9 (4.5–5.1)	13.6 (12.1–16.6)	9.2 (6.6–10.2)	6.5 (4.7–7.5)			
Sham	3.5 (3.3–3.7)	4.1 (3.7–4.4)	4.0 (3.7–4.4)	4.4 (4.0–4.7)			
$\text{Pvmes-}{\text{a}}_{\text{C}{\text{O}}_{2}}$, mmHg							
Placebo	5.8 (3.7–6.8)	18.4 (18.2–18.9)	17.6 (11.0–22.4)*	19.2 (9.3–19.2)*	<0.001	0.01	0.05
Dobutamine	5.8 (3.8–6.4)	20.3 (17.5–23.4)	7.0 (5.7–11.1)	5.1 (3.7–5.9)			
Sham	6.3 (4.8–7.7)	3.6 (3.5–3.7)	3.6 (3.5–3.7)	4.6 (2.7–6.5)			
$\text{Ptis-}{\text{a}}_{\text{C}{\text{O}}_{2}}$, mmHg							
Placebo	10.4 (6.4–11.1)	32.0 (25.9–34.0)	23.4 (23.3–30.6)	33.4 (24.7–34.8)*	<0.001	0.01	0.01
Dobutamine	11.1 (4.5–14.0)	30.7 (28.5–34.7)	20.4 (19.4–24.3)	16.0 (14.2–16.9)			
Sham	9.5 (8.5–10.4)	6.4 (3.9–8.8)	6.8 (5.2–8.4)	13.1 (12.4–13.7)			
Lactate-art, mmol/l							
Placebo	1.8 (1.7–2.3)	7.4 (7.1–7.8)	8.3 (6.7–9.9)*	8.9 (8.4–9.3)*	<0.001	0.03	0.02
Dobutamine	1.9 (1.9–2.1)	7.5 (7.5–8.1)	4.4 (3.8–7.4)	2.3 (2.1–3.9)			
Sham	1.4 (1.1–1.7)	0.9 (0.8–1.0)	1.1 (1.0–1.2)	1.2 (0.7–1.8)			
Lactate-mes, mmol/l							
Placebo	2.6 (2.0–2.6)	8.2 (7.9–8.5)	8.9 (5.6–12.2)	7.3 (4.6–10.3)*	<0.001	0.02	0.03
Dobutamine	2.1 (1.8–2.2)	7.6 (7.3–8.2)	6.4 (3.8–7.1)	4.2 (3.4–4.8)			
Sham	1.6 (1.2–2.0)	1.0 (1.0–1.1)	1.2 (1.1–1.3)	1.1 (0.8–1.4)			
$\text{S}{\text{V}}_{{\text{O}}_{2}}$, %							
Placebo	67.6 (61.4–70.3)	68.8 (55.6–72.5)	73.7 (73.4–74.8)	77.5 (71.4–77.8)	0.14	0.19	0.22
Dobutamine	62.5 (53.0–71.1)	62.0 (61.2–71.7)	66.1 (62.6–66.4)	65.8 (64.5–66.0)			
Sham	71.4 (69.8–71.9)	72.7 (68.3–77.1)	65.9 (65.8–66.0)	77.1 (74.5–79.6)			
$\text{Sv}{\text{mes}}_{{\text{O}}_{2}}$, %							
Placebo	74.4 (72.3–75.0)	34.4 (32.5–36.0)	38.4 (35.1–52.7)*	52.2 (48.9–57.2)*	<0.001	0.08	0.03
Dobutamine	75.5 (74.9–78.0)	32.0 (30.5–32.5)	58.7 (45.0–60.5)	67.5 (64.4–68.4)			
Sham	73.9 (72.3–75.6)	67.5 (66.1–68.8)	68.5 (67.0–70.0)	70.8 (69.9–71.6)			
Do_2_, ml·kg^−1^·min^−1^							
Placebo	17.0 (14.1–18.9)	13.4 (12.5–15.1)	17.1 (15.8–21.9)	18.4 (17.7–18.7)	0.01	0.84	0.56
Dobutamine	14.5 (13.5–17.3)	13.9 (10.9–15.0)	18.6 (12.2–19.3)	18.0 (15.4–20.0)			
Sham	12.5 (12.4–12.5)	12.9 (12.3–13.5)	13.0 (12.3–13.6)	13.0 (12.1–13.8)			
V̇o_2_, ml·kg^−1^·min^−1^							
Placebo	5.9 (4.4–7.0)	5.8 (4.9–6.2)	8.2 (7.8–8.3)	8.9 (7.1–8.9)	0.003	0.38	0.18
Dobutamine	5.6 (4.7–5.7)	6.0 (4.7–6.5)	7.3 (6.3–7.9)	6.2 (6.1–7.1)			
Sham	4.2 (4.0–4.4)	4.7 (4.7–4.8)	4.5 (4.2–4.8)	4.7 (4.4–5.0)			
Systemic $\text{E}{\text{R}}_{{\text{O}}_{2}}$							
Placebo	0.34 (0.30–0.40)	0.42 (0.37–0.46)	0.47 (0.41–0.48)	0.48 (0.40–0.55)	0.02	0.20	0.40
Dobutamine	0.36 (0.35–0.39)	0.40 (0.39–0.47)	0.41 (0.40–0.41)	0.40 (0.36–0.40)			
Sham	0.33 (0.32–0.35)	0.37 (0.35–0.38)	0.35 (0.34–0.35)	0.36 (0.36–0.37)			
Mesenteric $\text{E}{\text{R}}_{{\text{O}}_{2}}$							
Placebo	0.27 (0.27–0.29)	0.65 (0.64–0.65)	0.62 (0.47–0.63)*	0.44 (0.44–0.46)*	<0.001	0.04	0.02
Dobutamine	0.26 (0.23–0.26)	0.68 (0.64–0.68)	0.37 (0.37–0.49)	0.32 (0.25–0.35)			
Sham	0.27 (0.25–0.28)	0.33 (0.32–0.35)	0.32 (0.30–0.34)	0.31 (0.29–0.32)			
*Fluids/vasopressors*							
Cumulative fluids, ml							
Placebo		2,013 (1,977–2,237)	2,810 (2,643–2,877)	3,285 (3,218–3,339)	<0.001	0.90	0.85
Dobutamine		2,190 (2,112–2,376)	2,792 (2,790–3,116)	3,248 (3,150–3,644)			
Sham		1259 (1200–1290)	1,559 (1,500–1,600)	1,973 (1,950–1,998)			
Norepinephrine, µg·kg^−1^·min^−1^							
Placebo		0.52 (0.42–0.53)	0.28 (0.25–0.30)	0.20 (0.18–0.24)	<0.001	0.94	0.44
Dobutamine		0.49 (0.45–0.58)	0.26 (0.24–0.32)	0.20 (0.20–0.22)			
Sham							

All data are presented as medians (25–75 percentiles); *n* = 6 (dobutamine), 6 (placebo), and 3 (sham) animals in each group. TS, time shock; T2H, 2 h after starting resuscitation; T6H, 6 h after starting resuscitation; HR, heart rate; CO, cardiac output; MAP, mean arterial pressure; CVP, central venous pressure; pH, arterial pH; $\text{P}{\text{a}}_{\text{CO}}{}_{{}_{2}}$, arterial CO_2_ partial pressure; $\text{Pv-}{\text{a}}_{\text{C}{\text{O}}_{2}}$, mixed venous-to-arterial CO_2_ difference; $\text{Pvmes-}{\text{a}}_{\text{C}{\text{O}}_{2}}$, mesenteric venous-to-arterial CO_2_ difference; $\text{Ptis-}{\text{a}}_{\text{C}{\text{O}}_{2}}$, tissue (jejunal mucosa)-to-arterial CO_2_ difference; Lactate art, arterial lactate; Lactate mes, mesenteric lactate; $\text{S}{\text{V}}_{{\text{O}}_{2}}$, mixed-venous O_2_ saturation; $\text{Sv}{\text{mes}}_{{\text{O}}_{2}}$, mesenteric-venous O_2_ saturation; Do_2_, systemic O_2_ delivery; V̇o_2_, systemic O_2_ consumption; systemic $\text{E}{\text{R}}_{{\text{O}}_{2}}$, systemic O_2_ extraction ratio; mesenteric $\text{E}{\text{R}}_{{\text{O}}_{2}}$, mesenteric O_2_ extraction ratio.

* SNK test, *P* < 0.05 for P vs. D group.

#### Microcirculatory blood flow.

**Fig. 2.**
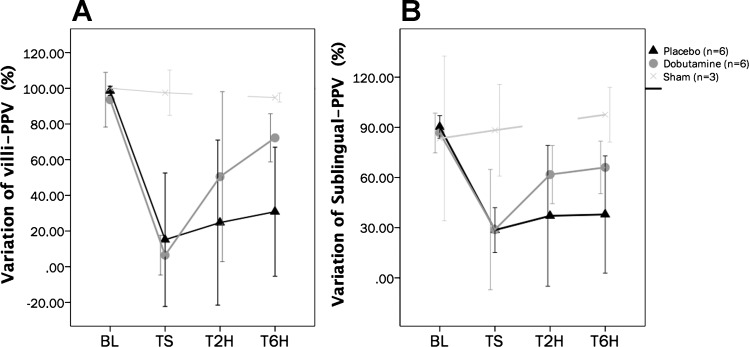
Time course of variations in the percentage of villi with predominantly well-perfused small vessels (%villi-PPV) and the percentage of small vessels perfused at sublingual mucosa (SL-PPV). *A*: %villi-PPV: repeated-measurements analysis, *P* < 0.001 for time effect; *P* = 0.02 for time-group interactions; *P* = 0.04 for intergroup differences. Student-Newman-Keuls (SNK) test, *P* < 0.05 for sham vs. P and sham vs. D groups at TS, T2H, and T6H; SNK test, *P* < 0.05 for P vs. D at T2H and T6H. *B*: SL-PPV: repeated-measures analysis, *P* < 0.001 for time effect; *P* = 0.02 for time-group interaction; *P* = 0.01 for intergroup differences. SNK test, *P* < 0.05 for sham vs. P and sham vs. D groups at TS, T2H, and T6H; *P* < 0.05 for P vs. D at T2H and T6H). No. of animals is as follows: dobutamine (*n* = 6), placebo (*n* = 6), and sham (*n* = 3).

**Fig. 3.**
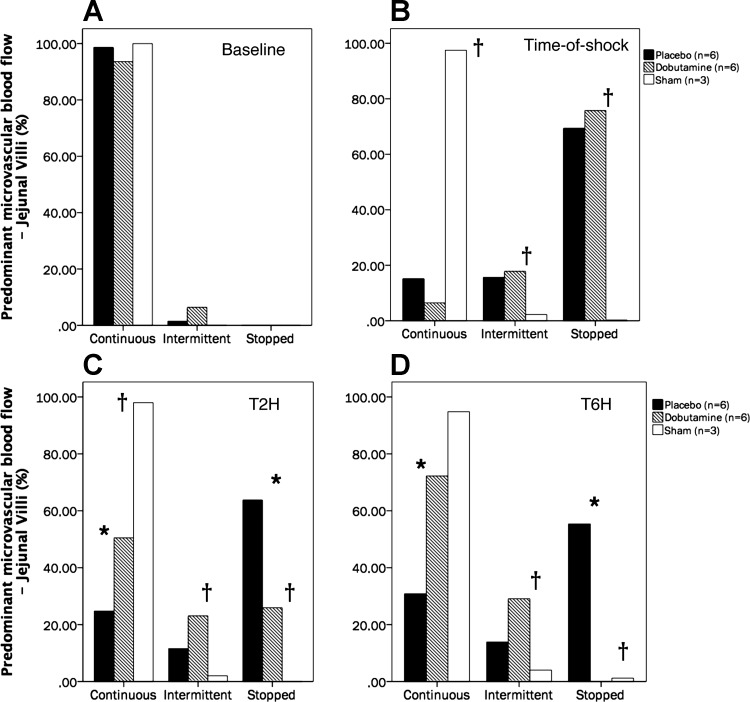
Predominant type of microvascular flow in the jejunal villi at each measurement time. Percentage distribution of predominant microvascular blood flow in jejunal villi capillaries at baseline (*A*), TS (*B*), T2H (*C*), and T6H (*D*). †*P* < 0.05 between sham vs. dobutamine and placebo groups. *P < 0.05 between dobutamine vs. placebo groups. No. of animals is as follows: dobutamine (*n* = 6), placebo (*n* = 6), and sham (*n* = 3).

**Fig. 4.**
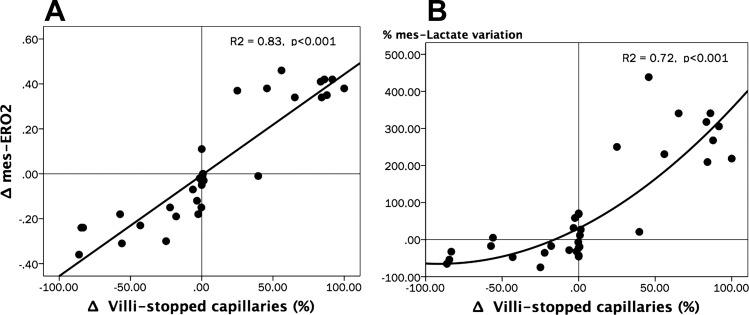
Relationships between the variations of the percentage of villi with stopped capillary flow (Δvilli-stopped capillaries), mesenteric O_2_ extraction ratio (Δ$\text{mes-E}{\text{R}}_{{\text{O}}_{2}}$), and percentage changes of mesenteric lactate levels (%mes-Lactate variation). *A*: scatterplot depicting the relationships between Δvilli-stopped capillaries vs. Δ$\text{mes-E}{\text{R}}_{{\text{O}}_{2}}$. Spearman Rho test, *R*^2^ = 0.83, *P* < 0.001. *B*: scatterplot depicting the relationships between Δvilli-stopped capillaries vs. %mes-Lactate variation. Spearman Rho test, *R*^2^ = 0.72, *P* < 0.001. All absolute or percentage variations (Δ) were calculated as the change between actual and its immediately preceding value (*A* and *B*).

Complete intestinal and sublingual microcirculatory parameters are presented in Table 2. The time course of the proportion of jejunal villi with normal-perfused vessels (%Villi-PPV) was significantly different between groups (repeated-measures analysis, *P* = 0.02 for time × group interactions and *P* = 0.04 for intergroup differences; SNK test, *P* < 0.05 for P vs. D at T2H and T6H) (Fig. 2). The percentage of small vessels perfused at the sublingual mucosa (SL-PPV) exhibited similar variations to villi-PPV, with significant improvement in the D group, although without attaining complete normalization at the end of the experiment (Fig. 2). An excess of stopped vessels at the intestinal mucosa explained the decreasing %villi-PPV at TS in the P and D groups (Fig. 3). Thereafter, the D group exhibited significant and progressive improvement of microcirculation at the jejunal-villi, moving from stopped to intermittent and continuous flows, thus improving functional capillary density and decreasing microvascular blood flow heterogeneity (Fig. 3), although without attaining complete normalization at T6H. Dynamic changes in the proportion of capillaries with stopped flow were significantly related to variations in $\text{mes-E}{\text{R}}_{{\text{O}}_{2}}$ (Spearman Rho test, *R*^2^ = 0.83, *P* < 0.001) and lactate levels (Spearman Rho test, *R*^2^ = 0.72, *P* < 0.001) (Fig. 4). Meanwhile, jejunal serosa and sublingual mucosa exhibited similar variations to those observed at the jejunal-villi, with severely decreased PPV at TS, followed by significant recovery in the D group (Table 2). Such alterations were paralleled by decreased functional capillary density and increasing microvascular blood flow heterogeneity at both the jejunal serosa and sublingual mucosa (Table 2). Examples of the intestinal microcirculatory blood flow images captured by the SDF technique during the development and resuscitation phase of the experiment are presented as supplemental data [baseline (Supplemental Videos 1A–1B); TS (Supplemental Videos 2A–2B), and dobutamine at T6H (Supplemental Videos 3A–3B) (Supplemental videos for this article can be found on the journal website.)]. Our coefficient of variability of the determination of one video sequence ranged from 2.9 to 6.4% (intraobserver) and from 3.8 to 6.2% (interobserver) for the total number of vessels and from 1.9 to 4.5% (intraobserver) and from 3.4 to 6.8% (interobserver) for the proportion of perfused vessels (all sizes).

**Table 2.** Microcirculatory blood flow parameters

					Intragroup Difference		
	Baseline	TS	T2H	T6H	Time effect	Time × group effect	Intergroup Difference
Villi-PPV, %							
Placebo	100.0 (97.5–100.0)	0.0 (0.0–6.8)	6.9 (0.0–28.9)*	18.8 (17.0–31.8)*	<0.001	0.02	0.04
Dobutamine	98.5 (98.0–100.0)	0.0 (0.0–13.9)	48.9 (16.7–76.5)	79.0 (67.4–79.6)			
Sham	97.5 (96.5–98.5)	97.5 (96.5–98.5)		94.8 (94.6–95.0)			
Ser-PPV, %							
Placebo	97.4 (90.1–99.7)	31.2 (16.7–37.8)	34.3 (22.6–49.2)	32.7 (6.5–58.7)*	<0.001	0.01	0.04
Dobutamine	100.0 (87.9–100.0)	16.7 (0.0–20.8)	45.4 (7.4–45.7)	85.7 (75.7–91.5)			
Sham	68.7 (67.8–69.6)	89.0 (88.5–89.4)		97.8 (95.6–100.0)			
Ser-FCD, vessels/mm^2^							
Placebo	6.8 (5.6–7.9)	1.3 (0.4–2.2)	2.0 (1.1–2.3)	2.5 (2.1–2.7)*	<0.001	0.12	0.03
Dobutamine	6.3 (5.9–8.8)	0.9 (0.0–1.2)	2.1 (0.4–2.4)	5.2 (3.5–5.6)			
Sham	5.5 (5.3–5.6)	6.8 (6.7–6.8)		6.3 (5.2–7.4)			
Ser-HI							
Placebo	0.2 (0.0–0.2)	1.5 (0.8–2.0)	1.0 (1.0–1.5)*	2.0 (1.5–2.6)*	0.03	0.12	0.03
Dobutamine	0.0 (0.0–0.3)	2.0 (0.0–2.8)	0.5 (0.2–0.6)	0.1 (0.0–0.3)			
Sham	0.7 (0.3–1.1)	0.3 (0.2–0.4)		0.3 (0.0–0.6)			
SL-PPV, %							
Placebo	87.0 (85.9–95.7)	31.8 (21.2–32.1)	34.5 (8.2–67.2)*	22.8 (22.7–67.0)*	<0.001	0.02	0.01
Dobutamine	86.2 (81.0–95.1)	20.7 (13.2–29.9)	68.3 (47.5–71.1)	69.1 (58.2–70.3)			
Sham	83.3 (79.5–87.2)	88.3 (86.1–90.4)		97.6 (96.3–98.9)			
SL-FCD, vessels/mm^2^							
Placebo	7.0 (6.9–7.1)	2.5 (1.6–3.0)	2.8 (0.5–3.8)*	2.1 (1.9–4.5)*	<0.001	0.11	0.04
Dobutamine	6.5 (6.0–6.9)	1.7 (0.8–2.2)	4.6 (4.0–5.7)	4.6 (4.5–5.7)			
Sham	7.6 (7.4–7.8)	9.1 (8.9–9.3)		10.9 (10.9–11.0)			
SL-HI							
Placebo	0.3 (0.1–0.4)	2.1 (2.0–3.1)	1.5 (0.8–2.3)	1.7 (1.1–2.5)*	<0.001	0.02	0.04
Dobutamine	0.8 (0.8–0.9)	1.5 (1.5–2.6)	0.9 (0.8–1.2)	0.9 (0.4–1.0)			
Sham	0.4 (0.3–0.4)	0.3 (0.2–0.3)		0.1 (0.1–0.1)			

All data are presented as medians (25–75 percentiles); *n* = 6 (dobutamine), 6 (placebo), and 3 (sham) animals in each group. Villi-PPV, proportion of jejunal-villi with well-perfused vessels; Ser-PPV, percentage of small vessels perfused at jejunal serosa; Ser-FCD, functional capillary density at jejunal serosa; Ser-HI, heterogeneity index at jejunal serosa; SL-PPV, percentage of small vessels perfused at sublingual mucosa; SL-FCD, functional capillary density at sublingual mucosa; SL-HI, heterogeneity index at sublingual mucosa.

* SNK test, *P* < 0.05 for P vs. D group.

#### $\text{E}{\text{R}}_{{\text{O}}_{2}}$ and its relationships with microcirculatory blood flow.

**Fig. 5.**
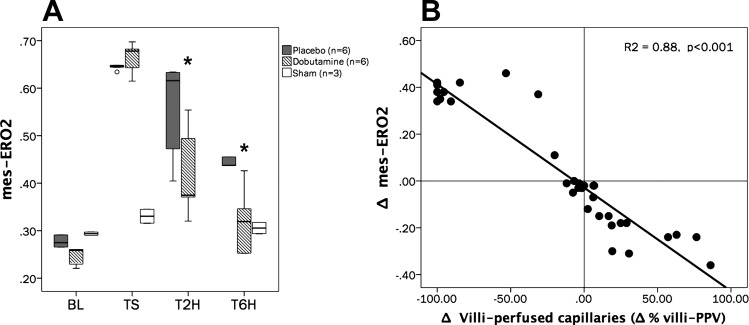
Time course of mesenteric O_2_ extraction ratio ($\text{mes-E}{\text{R}}_{{\text{O}}_{2}}$) and its relationships with variations in villi-PPV. *A*: time course of $\text{mes-E}{\text{R}}_{{\text{O}}_{2}}$. Repeated measurements analysis, *P* = 0.04 for time-group interactions; *P* = 0.02 for intergroup differences. *P < 0.05 for P vs. D groups. *B*: scatterplot for Δ% villi-PPV vs. Δ$\text{mes-E}{\text{R}}_{{\text{O}}_{2}}$. (Spearman Rho test, *R*^2^ = 0.88, *P* < 0.001). Δ% villi-PPV and Δ$\text{mes-E}{\text{R}}_{{\text{O}}_{2}}$ were calculated as the variation between actual and its immediately preceding value (*B*). No. of animals is as follows: dobutamine (*n* = 6), placebo (*n* = 6), and sham (*n* = 3).

$\text{mes-E}{\text{R}}_{{\text{O}}_{2}}$ showed a significant increase in both P and D groups at TS [0.65 (0.64–0.65) vs. 0.68 (0.64–0.68) for P and D groups, respectively; Mann-Whitney Rank sum test, *P* > 0.05]. Despite significant improvement in $\text{mes-E}{\text{R}}_{{\text{O}}_{2}}$ after starting resuscitation in both experimental groups, the slope of $\text{mes-E}{\text{R}}_{{\text{O}}_{2}}$ recovery was significantly steeper in the D group (repeated-measures analysis, *P* = 0.04 for time × group interaction and *P* = 0.02 for intergroup differences. SNK test, *P* < 0.05 for sham vs. P and D at TS; *P* < 0.05 for P vs. D at T2H and T6H) (Fig. 5*A*). Variations of villi-PPV during shock development and resuscitation depicted a very good agreement with variations in $\text{mes-E}{\text{R}}_{{\text{O}}_{2}}$ (Spearman Rho test, *R*^2^ = 0.88, *P* < 0.001) (Fig. 5*B*).

#### Systemic, regional, and local CO_2_ to arterial differences.

**Fig. 6.**
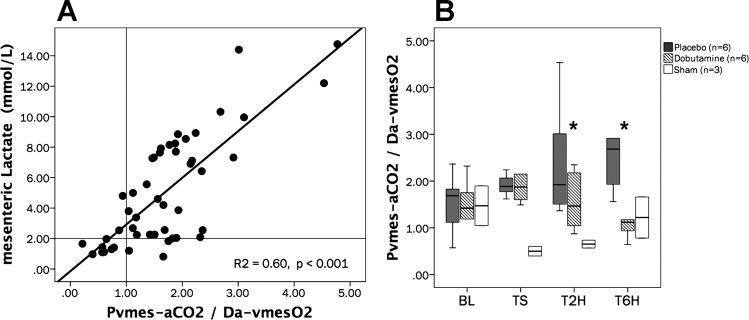
Relationships between $\text{Pvmes-}{\text{a}}_{\text{C}{\text{O}}_{2}}$/$\text{Ca}{\text{-vmes}}_{{\text{O}}_{2}}$ ratios vs. mesenteric-venous lactate levels and time course of $\text{Pvmes-}{\text{a}}_{\text{C}{\text{O}}_{2}}$/$\text{Ca}{\text{-vmes}}_{{\text{O}}_{2}}$ ratios. *A*: scatterplot depicting the relationships between the mesenteric venous-arterial CO_2_ to arterial-venous O_2_ pressure differences ratio ($\text{Pvmes-}{\text{a}}_{\text{C}{\text{O}}_{2}}$/$\text{Ca}{\text{-vmes}}_{{\text{O}}_{2}}$ ratio) vs. mesenteric-venous lactate levels. Spearman Rho test, *R*^2^ = 0.60, *P* < 0.001. *B*: time course of the mesenteric venous-arterial CO_2_ to arterial-venous O_2_ pressure differences ($\text{Pvmes-}{\text{a}}_{\text{C}{\text{O}}_{2}}$/$\text{Da}{\text{-vmes}}_{{\text{O}}_{2}}$). Repeated-measures analysis, *P* = 0.32 for time effect; *P* = 0.06 for time-group interaction; *P* = 0.05 for intergroup differences. *P < 0.05 for D vs. P groups (*B*). No. of animals is as follows: dobutamine (*n* = 6), placebo (*n* = 6), and sham (*n* = 3).

Variations in the tissue-to-arterial CO_2_ difference ($\text{Pti-}{\text{a}}_{\text{C}{\text{O}}_{2}}$) agreed with regional changes ($\text{Pmes-}{\text{a}}_{\text{C}{\text{O}}_{2}}$) and these, in turn, with systemic venous-to-arterial CO_2_ differences ($\text{P}\bar{\text{v}}\text{-}{\text{a}}_{\text{C}{\text{O}}_{2}}$) (Table 1). $\text{Pti-}{\text{a}}_{\text{C}{\text{O}}_{2}}$ and $\text{Pmes-}{\text{a}}_{\text{C}{\text{O}}_{2}}$ exhibited a good agreement with villi-PPV (Spearman-Rho test, *R*^2^ = 0.69 and 0.63, respectively, *P* < 0.001), and showed a more discrete agreement with Ser-PPV (Spearman-Rho test, *R*^2^ = 0.48 and 0.47, respectively, *P* < 0.001). Meanwhile, mesenteric venous-arterial CO_2_ to arterial-venous O_2_ pressure differences ratio ($\text{Pvmes-}{\text{a}}_{\text{C}{\text{O}}_{2}}$/$\text{Ca}{\text{-v}}_{{\text{O}}_{2}}$ ratio) was well correlated with mesenteric lactate levels (Spearman-Rho test, *R*^2^ = 0.60, *P* < 0.001), suggesting the appearance and reversal of anaerobic metabolism during shock development and the resuscitation phase (in the D group), respectively (Fig. 6*A*). Notably, the time course of the $\text{Pvmes-}{\text{a}}_{\text{C}{\text{O}}_{2}}$-to-$\text{Ca}{\text{-v}}_{{\text{O}}_{2}}$ ratio was significantly different in experimental groups, showing a significant decrease in the D group at T6H (Fig. 6*B*).

#### Relationships between variations of lactate levels, regional O_2_ extraction ratio, and jejunal microcirculatory blood flow.

**Fig. 7.**
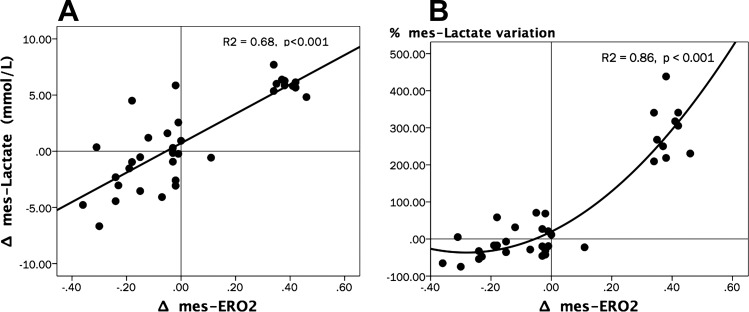
Relationships between variations of Δ$\text{mes-E}{\text{R}}_{{\text{O}}_{2}}$, Δmes-Lactate, and %mes-Lactate variation. *A*: scatterplot depicting the relationships between Δ$\text{mes-E}{\text{R}}_{{\text{O}}_{2}}$ vs. Δmes-Lactate. Spearman Rho test, *R*^2^ = 0.68, *P* < 0.001. *B*: scatterplot depicting the relationships between Δ$\text{mes-E}{\text{R}}_{{\text{O}}_{2}}$ vs. %mes-Lactate variation. Spearman Rho test, *R*^2^ = 0.86, *P* < 0.001. All percentage or absolute variations (Δ) were calculated as the variation between actual and its immediately preceding value (*A* and *B*).

We observed significant increases in arterial and venous mesenteric lactate levels at TS. Models subjected to dobutamine infusion showed a significant decrease at T2H and T6H in both arterial lactate (repeated-measures analysis, *P* ≤ 0.001 for the time effect; *P* = 0.03 for time × group interaction; and *P* = 0.02 for intergroup differences; SNK test, *P* < 0.05 for P vs. D at T2H and T6H) and venous-mesenteric lactate (repeated-measures analysis, *P* ≤0.001 for the time effect, *P* = 0.02 for time × group interaction, and *P* = 0.03 for intergroup differences; SNK test, *P* < 0.05 for P vs. D at T2H and T6H) levels, whereas lactate levels remained high in the placebo group (Table 1). Variations in $\text{mes-E}{\text{R}}_{{\text{O}}_{2}}$ were well correlated with changes in both absolute venous lactate values and the percentage of variation of venous lactate (using as reference the immediately preceding value) (Fig. 7).

## DISCUSSION

Our results suggest that variations in the distribution of intestinal microcirculatory blood flow at the jejunal-villi during the development of, and resuscitation from, septic shock are closely related to changes in regional O_2_ extraction ratios and these, in turn, to variations in mesenteric lactate levels. Although cardiac output and systemic O_2_ delivery evolved similarly in both the D and P groups, the heterogeneity of microcirculatory blood flow at the jejunal mucosa and serosa was significantly reversed after dobutamine infusion, which was accompanied by decreases in mesenteric O_2_ extraction ratio and mesenteric lactate levels.

Variations in $\text{E}{\text{R}}_{{\text{O}}_{2}}$ denote adaptive cellular responses to O_2_ availability. Indeed, experimental models during early stages of sepsis demonstrated up to a threefold increase in the capillary $\text{E}{\text{R}}_{{\text{O}}_{2}}$ in normal perfused muscle, which intuitively suggest that increases in $\text{E}{\text{R}}_{{\text{O}}_{2}}$ should reflect the maximization of such adaptation to hypoxic tissue challenges (12). However, the assumption that tissue oxygenation can be preserved by maintaining its blood supply is derived from models that presume uniformly perfused capillaries. Conversely, septic shock is characterized by increased heterogeneity of microcirculatory blood flow, which implies the presence of zones of tissue receiving an adequate perfusion through capillaries with continuous flow in close proximity with zones with no microvascular perfusion due to capillaries with stopped flow (12), which leads to inhomogeneity of O_2_ distribution and, therefore, to abnormal cellular respiration, such as has been observed using in vivo NADH videofluorimetry techniques (22). We observed a severe decline in the proportion of small vessels perfused at both the jejunal and sublingual mucosa at the time of shock with a subsequent increase in blood flow heterogeneity and decrease in functional capillary density. Such as mathematical models have suggested, heterogeneity of O_2_ delivery should respond in part for tissue O_2_ derangements in peripheral tissues (49). Indeed, experimental evidence from ex vivo intestinal pieces after endotoxin infusion suggests that the heterogeneity of gut capillary transit times is related to impairment of gut O_2_ extraction (21). However, the static concept of such experiments (21) makes it difficult to understand the relationships between microvascular blood flow heterogeneity and O_2_ utilization during the development and resuscitation of septic shock. Conversely, the dynamic nature of our experiment reveals that variations in the percentage of well-perfused capillaries at jejunal villi (villi-PPV) are closely related with dynamic changes in mesenteric $\text{E}{\text{R}}_{{\text{O}}_{2}}$ and these, in turn, with variations in mesenteric lactate values. Mathematical modeling of O_2_ demand (do_2_) to O_2_ supply (qo_2_) distributions (do_2_/qo_2_) demonstrates that increases in its relative dispersion lead to a nonlinear decrease in the critical O_2_ extraction ratio (49). Thus, deviations of do_2_ vs. do_2_/qo_2_ distribution “to the right” (i.e., >1.0) suggest the appearance of anaerobic metabolism even at the same total O_2_ transport (Do_2_) values, i.e., attainment of anaerobic thresholds at lower critical $\text{E}{\text{R}}_{{\text{O}}_{2}}$ and Do_2_ values (49). In our experiment, rises in regional O_2_ extraction ratios were accompanied by increases in both total (crude) and percentage of variation in mesenteric lactate levels, which reinforces the notion that alterations in do_2_/qo_2_ distributions attained during our experiment were enough to overcome anaerobic metabolism thresholds previously reported in experimental conditions (21, 27, 28, 38).

We observed an increased number of stopped vessels at the time of shock coinciding with the higher $\text{mes-E}{\text{R}}_{{\text{O}}_{2}}$ and mesenteric lactate levels. We hypothesized that increases in the proportion of capillaries with stopped blood flow could overcome the capacity of O_2_ delivery from capillaries with normal flow, thus leading to tissue O_2_ supply limitation. Thus, mesenteric-$\text{E}{\text{R}}_{{\text{O}}_{2}}$ was increased to the extent that functional capillary density declined with the subsequent widening of O_2_ diffusion distances. As a result, anaerobic metabolism was triggered, leading to increases of regional lactate levels, thus suggesting a key role for the heterogeneous stoppage of individual capillaries on abnormal O_2_ utilization during very early stages of septic shock. Previous mathematical modeling revealed that, for three forms of progressive hypoxia (anemic, stagnant, and hypoxic), critical total O_2_ transport is quite similar, as long as intercapillary distances are <80 µm (39). Nevertheless, at higher intercapillary distances (i.e., at lower functional capillary densities), O_2_ consumption (V̇o_2_) becomes dependent from total O_2_ transport (Do_2_) for a wide range of values (39), suggesting that increments in heterogeneity of microvascular blood flow lead to do_2_/qo_2_ mismatching (which is reflected by low critical $\text{E}{\text{R}}_{{\text{O}}_{2}}$ values), thus indicating an impairment of O_2_ extraction ability (49). In our study, models receiving dobutamine showed a gradual replacement of stopped with intermittent and normal flows, which improved the functional capillary density and led to a parallel reduction in $\text{mes-E}{\text{R}}_{{\text{O}}_{2}}$ and regional lactate levels. This phenomenon suggests that, as microvascular blood flow became more homogeneous, tissues should exhibit better do_2_/qo_2_ distributions with the subsequent reversion of anaerobic metabolism. Some studies have proposed that the heterogeneous cessation of flow in individual capillaries could determine O_2_ supply dependence during septic shock (11), whereas mathematical modeling of this phenomenon suggests that oxygenation derangements are more severe when capillary blood flow is totally stopped than when it is intermittent (16), which also agrees with our results.

Dobutamine has shown contradictory results on macrovascular splanchnic (2, 3, 13, 33, 35, 37, 39, 47), total intestinal microvascular blood flow (19, 29), and sublingual microcirculation (7, 12, 18). Conversely, information about the effect of dobutamine on microcirculatory blood flow distribution at intestinal villi during sepsis or endotoxemia has been limited but favorable (42). Interestingly, mesenteric arterial blood flow can be relatively dissociated from microvascular blood flow (46), and misdistribution of blood flow within the intestinal wall might be observed at both hypodynamic and resuscitated phases of septic shock (20). We also observed this apparent dissociation between macro- and microhemodynamics during the resuscitation phase, as has been noted in previous studies (8, 30), but we did not observe discrepancies for serosal-to-mucosal microvascular blood flow distribution during development of shock nor during the resuscitation phase. Effects of dobutamine on villi microcirculation could be mediated by direct adrenergic stimulation (43), nitric oxide generation (24), decreased expression of endothelial E-selectin (45), or inhibition of nuclear factor-κB (25), although the exact mechanisms through which dobutamine exerted its effect on the microcirculatory blood flow go beyond the scope of our study.

The microcirculatory alterations observed in our experiment were accompanied by increases in jejunal-mucosa and regional mesenteric-to-arterial CO_2_ differences, and these were, in turn, reflected by the accumulation of systemic-venous CO_2_. Interestingly, the grade of recovery of microcirculation was mirrored by decreases in $\text{Pti-}{\text{a}}_{\text{C}{\text{O}}_{2}}$ and $\text{Pmes-}{\text{a}}_{\text{C}{\text{O}}_{2}}$, suggesting a link between microcirculatory blood flow and tissue or venous CO_2_ accumulation, respectively, as has recently been suggested by observations in human septic shock (31). Additionally, we observed an increased mesenteric-venous CO_2_-to-arterial venous O_2_ differences ratio ($\text{Pvmes-}{\text{a}}_{\text{C}{\text{O}}_{2}}$/$\text{Ca}{\text{-vmes}}_{{\text{O}}_{2}}$ ratio) during the development of shock in both experimental groups, evolving in parallel with regional lactate accumulation. Such increased CO_2_-to-O_2_ relationship has been proposed as reflection of anaerobic metabolism, since under aerobic steady-state conditions, CO_2_ production (V̇co_2_) approximates O_2_ consumption (V̇o_2_), and, consequently, the mixed venous-to-arterial CO_2_ content difference ($\text{C}\bar{\text{v}}\text{-}{\text{a}}_{\text{C}{\text{O}}_{2}}$) approximates the arterial-to-mixed-venous O_2_ content difference ($\text{Ca-}{\bar{\text{v}}}_{{\text{O}}_{2}}$). Accordingly, the V̇co_2_-to-V̇o_2_ ratio (i.e., the respiratory quotient) should not be higher than 1.0, whereby nonsymmetric decreases in V̇o_2_ and V̇co_2_ with subsequent rises in the respiratory quotient could reflect nonaerobic CO_2_ generation (30, 32). Importantly, models subjected to dobutamine infusion in our experiment showed a progressive improvement in microvascular blood flow distribution that was in turn related to decreases in the $\text{Pvmes-}{\text{a}}_{\text{C}{\text{O}}_{2}}$/$\text{Ca}{\text{-vmes}}_{{\text{O}}_{2}}$ ratio (a regional surrogate of the V̇co_2_/V̇o_2_ ratio), suggesting the reversal of anaerobic metabolism while mesenteric lactate levels also decreased.

It could be argued that the thermogenic effects induced by dobutamine could explain the increases in the O_2_ extraction ratio in our experiment (15, 17). V̇o_2_ responses to prostacyclin (6) and nitroprusside (5) are, however, quite similar to the responses to dobutamine in septic patients and healthy volunteers (36). Additionally, the thermogenic effects of catecholamines are related to progressively increased doses, a situation that is avoided when low fixed doses are used (as in our study).

Our model had limitations. First, we focused on microcirculatory blood flow alterations while resuscitation was guided by macrohemodynamic variations, as occurs during resuscitation from human septic shock. Nonetheless, the macrohemodynamics evolved similarly in the dobutamine and placebo groups, suggesting that the effects of dobutamine were mediated at the microcirculatory level. Second, our institutional Animal Research Committee suggested limiting the total number of models, including those used during the preexperimental standardization phase, with the result that we included only three animals in the sham group. Admittedly, some differences were observed in certain macrohemodynamic variables at baseline when comparing the sham animals with the two experimental groups. The inclusion of a higher number of sham models would not, however, have changed our final results, since microcirculatory flow variables were almost identical at baseline in the sham, dobutamine, and placebo groups, whereas macrohemodynamics evolved in parallel in the two experimental groups. Third, our experiment reproduces a severe hypodynamic septic shock, which could distance our results from usual clinical observations. Our model does, however, closely recreate the initial phases of nonresuscitated septic shock with a complete spectrum of enteric pathogens, with immunological and macrohemodynamic disturbances occurring gradually. Fourth, we did not measure variations of arterial mesenteric blood flow, which precludes the analysis of mesenteric-Do_2_. However, alterations in microvascular blood flow distribution cannot be predicted from macrohemodynamics or even from total microcirculatory flow (i.e., by laser Doppler techniques). Thus, measurements of total microvascular blood flow and its distribution (i.e., estimation of blood flow heterogeneity) could be more, or at least, as relevant as isolated mesenteric arterial blood flow measurements. Furthermore, although indirect, the proportional decrease in mesenteric lactate levels in our experiment provides a strong suggestion that the increase in microvascular perfusion was associated with an improved metabolism associated with better O_2_ utilization.

In conclusion, variations in the heterogeneity of microcirculatory blood flow at the jejunal mucosa are closely linked to regional splanchnic O_2_ extraction ratios and mesenteric lactate levels, thus suggesting a key role for microvascular blood flow distribution on O_2_ utilization in septic shock. Low fixed doses of dobutamine can decrease such flow heterogeneity, promoting O_2_ metabolism recovery.

## GRANTS

This work was supported by Tecnoquímicas (Colombia)—Centro Investigaciones Clínicas, Fundación Valle del Lili (Colombia) (CIC 001) and Universidad ICESI (Colombia) (IP-FO-01).

## DISCLOSURES

No conflicts of interest, financial or otherwise, are declared by the authors.

## AUTHOR CONTRIBUTIONS

G.A.O.-T. conceived and designed research; G.A.O.-T., A.F.G.M., G.J.E., W.F.B., H.J.M.N., J.D.V., E.Q., F.R., and A.M. performed experiments; G.A.O.-T., A.F.G.M., G.J.E., W.F.B., H.J.M.N., J.D.V., E.Q., F.R., A.M., C.A.A.D., A.B., G.H., and D.D.B. analyzed data; G.A.O.-T., A.F.G.M., G.J.E., E.Q., A.M., C.A.A.D., A.B., and G.H. interpreted results of experiments; G.A.O.-T. prepared figures; G.A.O.-T. and C.A.A.D. drafted manuscript; G.A.O.-T., A.F.G.M., G.J.E., W.F.B., H.J.M.N., J.D.V., E.Q., A.M., C.A.A.D., A.B., G.H., and D.D.B. edited and revised manuscript; G.A.O.-T., A.F.G.M., G.J.E., W.F.B., H.J.M.N., J.D.V., E.Q., F.R., A.M., C.A.A.D., A.B., G.H., and D.D.B. approved final version of manuscript.

## Supplementary Material

Video_1A.mov

Video_1B.mov

Video_2A.mov

Video_2B.mov

Video_3A.mov

Video_3B.mov

## Glossary

### Abbreviations

CO: Cardiac output
CVP: Central venous pressure
Do_2_: Systemic O_2_ delivery
FCD: Functional capillary density
HI: Heterogeneity index
Lactate-art: Arterial lactate
Lactate-mes: Mesenteric lactate
MAP: Mean arterial pressure
$\text{mes-E}{\text{R}}_{{\text{O}}_{2}}$: Mesenteric O_2_ extraction ratio
$\text{P}{\text{a}}_{\text{CO}}{}_{{}_{2}}$: Arterial CO_2_ partial pressure
$\text{P}{\text{a}}_{\text{O}}{}_{{}_{2}}$: Arterial O_2_ partial pressure
PAOP: Pulmonary artery occlusion pressure
pH: Arterial pH
PPV: Percentage of small vessels perfused
$\text{Ptis-}{\text{a}}_{\text{C}{\text{O}}_{2}}$: Tissue (jejunal mucosa)-to-arterial CO_2_ difference
$\text{Pti}{\text{s}}_{\text{C}{\text{O}}_{2}}$: Tissue (jejunal mucosa) CO_2_ pressure
$\text{Pv-}{\text{a}}_{\text{C}{\text{O}}_{2}}$: Mixed venous-to-arterial CO_2_ difference
$\text{Pv-}{\text{a}}_{\text{C}{\text{O}}_{2}}$/$\text{Da-}{\text{v}}_{{\text{O}}_{2}}$: Mixed venous-to-arterial CO_2_ to arterial-venous O_2_ pressure differences ratio
$\text{Pvmes-}{\text{a}}_{\text{C}{\text{O}}_{2}}$: Mesenteric venous-to-arterial CO_2_ difference
$\text{Pvmes-}{\text{a}}_{\text{C}{\text{O}}_{2}}$/$\text{Da-}{\text{v}}_{{\text{O}}_{2}}$: Mesenteric blood venous-to-arterial CO_2_ to arterial-venous O_2_ pressure difference ratio
$\text{Pvme}{\text{s}}_{\text{C}{\text{O}}_{2}}$: Mesenteric venous CO_2_ partial pressure
$\text{Pvme}{\text{s}}_{{\text{O}}_{2}}$: Mesenteric venous O_2_ partial pressure
$\text{Pvm}{\text{x}}_{{\text{O}}_{2}}$: Mixed-venous O_2_ partial pressure
$\text{Pvm}{\text{x}}_{\text{C}{\text{O}}_{2}}$: Mixed-venous CO_2_ partial pressure
Ser-FCD: Functional capillary density at jejunal serosa
Ser-HI: Heterogeneity index at jejunal serosa
Ser-PPV: Percentage of small vessels perfused at jejunal serosa
SL-FCD: Functional capillary density at sublingual mucosa
SL-HI: Heterogeneity index at sublingual mucosa
SL-PPV: Percentage of small vessels perfused at sublingual mucosa
$\text{S}{\text{v}}_{{\text{O}}_{2}}$: Mixed-venous O_2_ saturation
$\text{Syst-E}{\text{R}}_{{\text{O}}_{2}}$: Systemic O_2_ extraction ratio
TCD: Total capillary density
%Villi-PPV: Proportion of jejunal-villi with well-perfused vessels
V̇o_2_: Systemic O_2_ consumption
